# Antibiotic subclasses differentially perturb the gut microbiota in kidney transplant recipients

**DOI:** 10.3389/frtra.2024.1400067

**Published:** 2024-09-20

**Authors:** Hanbo Dong, Runzhe Li, Ni Zhao, Darshana M. Dadhania, Manikkam Suthanthiran, John R. Lee, Wodan Ling

**Affiliations:** ^1^ Division of Biostatistics, Department of Population Health Sciences, Weill Cornell Medicine, New York, NY, United States; ^2^ Department of Biostatistics, Johns Hopkins Bloomberg School of Public Health, Baltimore, MD, United States; ^3^ Division of Nephrology and Hypertension, Department of Medicine, Weill Cornell Medicine, New York, NY, United States; ^4^ Department of Transplantation Medicine, New York Presbyterian Hospital–Weill Cornell Medical Center, New York, NY, United States

**Keywords:** kidney transplantation, gut microbiota, antibiotic subclasses, microbial diversity, differentially abundant bacteria

## Abstract

**Introduction:**

The impact of antibiotics on the gut microbiota in kidney transplant recipients is not well characterized. In this study, we determine the impact of different subclasses of antibiotics on the gut microbiota in a cohort of 168 kidney transplant recipients.

**Methods:**

Gut microbiome profiling was performed on 510 fecal specimens using 16S rRNA gene sequencing of the V4-V5 hypervariable region. We classified fecal specimens by antibiotic exposure into 5 categories: Beta-lactam, Fluoroquinolone (FQ), Beta-lactam & FQ Group, Other Antibiotics, and No Antibiotic (No Abx). Mixed-effects regression models were utilized to identify changes in microbial diversity and in the centered log-ratio (CLR) transformed abundance of genera while adjusting for important covariates.

**Results:**

Antibiotic administration was associated with a significant decrease in the Shannon alpha diversity index, a decreased abundance of 11 taxa including *Eubacterium* and *Ruminococcus*, and an increased abundance of 16 taxa including *Enterococcus* and *Staphylococcus.* Exposure to Beta-lactam antibiotics was associated with an increased abundance of 10 taxa including *Enterococcus* and a decreased abundance of 5 taxa including *Eubacterium* while exposure to FQ antibiotics was associated with an increased abundance of 3 taxa and a decreased abundance of 4 taxa including *Ruminococcus*.

**Conclusions:**

Beta-lactam antibiotics and FQ antibiotics have a profound impact on the gut microbiota in kidney transplant recipients. Given the link of the gut microbiota to infectious complications, antibiotic associated changes in the microbiota may lead to an increased risk for further infections.

## Introduction

Antibiotics are the mainstay to treat bacterial infections in the kidney transplant population. Antibiotics, however, have a variety of off-target adverse toxicities, which range from self-limiting gastrointestinal side effects to nephrotoxicity and neurotoxicity. Recent studies have underscored the importance of antibiotics on disturbing the gut microbiota months after antibiotic usage (1). Antibiotics are also a risk factor for the development of opportunistic pathogens such as *Clostridioides difficile* infections. Recent data further suggest that gut microbial domination with known pathogens can be associated with the development of other serious infections. In an allogeneic stem cell transplant population, gut domination with *Enterococcus* is associated with a nine-fold increased risk of *Enterococcus* bacteremia (2), and gut domination with gram-negative bacteria is associated with gram-negative bacteremia (3). In kidney transplant recipients, we have found that the gut abundance of *Escherichia* is associated with the development of *Escherichia* urinary tract infection (4).

Antibiotic usage is common early after transplantation in kidney transplant recipients, either as prophylaxis or treatment for infections. However, few studies have examined the impact of antibiotic therapy in this immunocompromised patient population. Given the multiple classes of antibiotics usage, detailed statistical analyses accounting for the serial collection of specimens from the same subjects are important for understanding the impact of subclasses of antibiotics on the gut microbiota.

To address existing gaps in knowledge, we evaluate the impact of subclasses of antibiotics on the gut microbiota over time in a well characterized cohort of 168 kidney transplant recipients (dbGap reference phs001879.v2.p1).

## Methods

Full details of methods are described in the Supplemental Methods with a Supplemental Flowchart showing the study design and analysis and a Supplemental Glossary explaining the statistical terms used in the analysis.

### Patient cohort/gut microbiota profiling

We evaluated 168 kidney transplant recipients who provided 510 fecal specimens for microbiome profiling from August 2015 to November 2016. All participants gave written informed consent in this study which was approved by the Weill Cornell Institutional Review Board. We performed 16S rRNA gene sequencing of the V4-V5 hypervariable region on each of the fecal specimens, as previously reported in (4), using an Illumina MiSeq Instrument (250 base pair by 250 base pair). Bioinformatic analysis to assign taxonomy is described in the Supplemental Methods and in (4).

### Antibiotic class classifications

Antibiotic administrations, in addition to preoperative antibiotic prophylaxis and *Pneumocystis jirovecii* (PJP) prophylaxis within the first 120 days after transplantation, were recorded. We treated antibiotic administration as a time-ever event. For example, if a kidney transplant recipient had repeated measurements of gut microbiota at post-transplant day 30, 45, and 60 and antibiotic treatment at post-transplant day 40, the antibiotic exposures at the 3 measurements are then defined as No Abx, Abx, and Abx. We aggregated the antibiotic exposures for each recipient over the first 120 days after transplantation to obtain a recipient-level grouping of antibiotic administration.

We grouped the antibiotics received into three major classes: Beta-lactams, Fluoroquinolones, or Other (Supplementary Table S1). We also categorized the kidney transplant recipients into the following major groups: Abx Group, kidney transplant recipients who received additional antibiotics beyond PJP prophylaxis and preoperative antibiotic prophylaxis (*N* = 89) and No Abx Group, kidney transplant recipients who did not receive additional antibiotics (*N* = 79). The most frequent subclass of antibiotics included the beta-lactam class and the fluoroquinolone class. Therefore, within the Abx Group, we subcategorized the kidney transplant recipients into: Beta-lactam Group, kidney transplant recipients who received beta-lactam antibiotics (*N* = 30); Fluoroquinolone (FQ) Group, kidney transplant recipients who received fluoroquinolone antibiotics (*N* = 25); Beta-lactam & FQ Group, kidney transplant recipients who received both beta-lactam antibiotics and fluoroquinolone antibiotics (*N* = 25); and Other Abx Group, kidney transplant recipients who received other antibiotics but never received beta-lactam or fluoroquinolone antibiotics (*N* = 9). Kidney transplant recipients may have received other antibiotics in the Beta-lactam Group, FQ Group, and Beta-lactam and FQ Group but were defined in the respective group. We note that this recipient-level grouping only presents a landscape of cohort division but wasn't used for the longitudinal statistical analysis due to its coarsened granularity.

We further categorized the 510 fecal specimens into 5 groups by exposure to Beta-lactam antibiotics (*n* = 63), FQ antibiotics (*n* = 35), Beta-lactams & FQ antibiotics (*n* = 25), Other antibiotics (*n* = 25), and no antibiotics (*n* = 362). This specimen-specific definition of antibiotic exposure was utilized in the longitudinal statistical analyses.

### Statistical analyses

Microbiome data were aggregated to the genus level and rare taxa present in less than 10% of the 510 specimens were filtered out. The zero microbial counts were imputed by the geometric Bayesian multiplicative replacement method (5), and the resulting imputed data was normalized by centered log-ratio (CLR) transformation (6), which removes the compositionality constraint of relative microbiome abundances and maps the data into Euclidean space where standard statistical analysis such as linear mixed-effects models can be applied.

Patient-level categorical variables between antibiotics groups were compared using Fisher's exact test and continuous variables were analyzed using Wilcoxon rank sum test (two-group comparisons) or Kruskal-Wallis test (multi-group comparisons). We conducted mixed-effects regression models on the alpha and beta-diversity indices while adjusting for covariates. To identify differentially abundant taxa, we used linear mixed-effects models to regress the CLR transformed abundances of each taxon on the time-ever antibiotics use while adjusting for covariates, with the resulting *p*-values adjusted by the Benjamini-Hochberg procedure. Further details of the in-depth statistical analyses can be found in the Supplemental Methods.

### Data availability

All antibiotic administrations and microbiome data can be found in the database of Genotype and Phenotype (dbGaP) phs 001879.v2.p1. Local institutional review board approval will be needed to access the data.

## Results

### Study cohort characteristics

We evaluated the gut microbial profiles in the 510 fecal specimens from 168 kidney transplant recipients collected within the first 3 months of transplantation. Among the 168 kidney transplant recipients, 89 (53%) were exposed to antibiotics during the first 3 months after transplantation (Abx Group) and 79 (47%) were not (No Abx Group). Among the Abx Group recipients, 30 were classified in the Beta-lactam Group, 25 in the FQ Group, 25 in the Beta-lactam & FQ Group, and 9 were in the Other Abx Group. The full list of antibiotics and their classes is shown in Supplementary Table S1.

Demographical and transplant characteristics, at the patient level, are shown in Supplementary Table S2A. Preoperative cefazolin prophylaxis (*p*-value = 0.025) and TMP/SMX PJP prophylaxis (*p*-value = 0.004) were less frequent in the Abx Group than in the No Abx Group. Female sex (*p*-value = 0.020) and deceased donor transplantation (*p*-value = 0.002) were more frequent in the Abx Group than in the No Abx Group. Demographical and transplant characteristics, at the fecal specimen level, are shown in Supplementary Table S2B.

Differences in demographical and transplant characteristics among the subgroups of the Abx Group and the No Abx Group are shown in Supplementary Table S3A. Supplementary Table S3B represents differences in demographic and transplant characteristics at the fecal specimen level.

### Gut microbial diversity changes after antibiotic exposure

**Figure 1:**
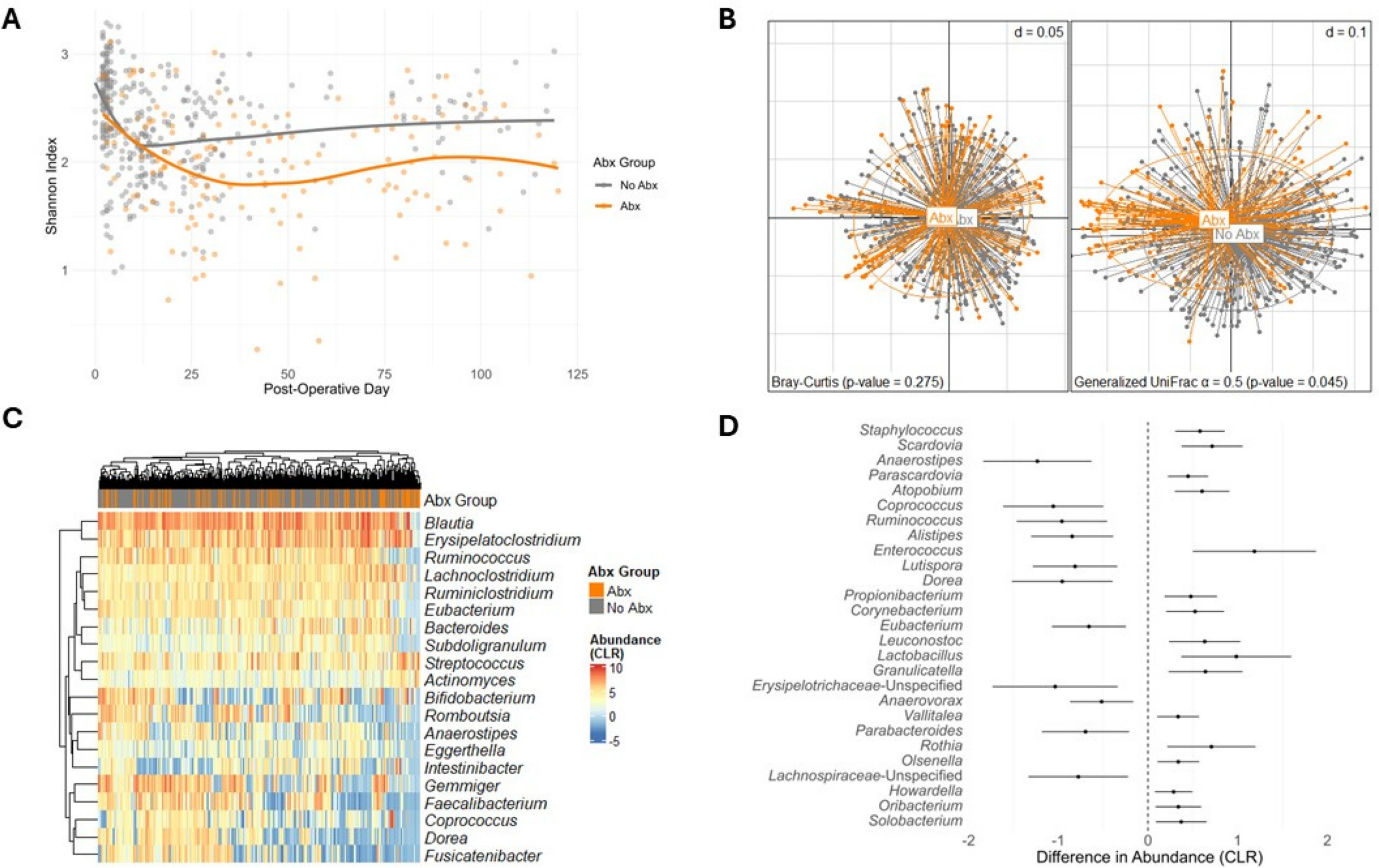
Analysis plots comparing exposure to antibiotics and No exposure to antibiotics. **(A)** Shannon Index over Post-Operative Days, comparing exposure to antibiotics (Abx) and no exposure to antibiotics (*p*-value < 0.001). Data points represent individual samples with the fitted smooth line showing the overall trend. **(B)** PCoA using Bray-Curtis dissimilarity (left) and generalized UniFrac (α = 0.5) dissimilarity (right), demonstrating beta diversity between exposure to antibiotics and no exposure to antibiotics [Bray-Curtis *p*-value = 0.275; generalized UniFrac (α = 0.5) *p*-value = 0.045]. Points represent individual samples and are connected to the group centroids. **(C)** Heatmap of CLR transformed abundances of the 20 most common taxa, clustered by hierarchical grouping, contrasting exposure to antibiotics with no exposure to antibiotics. Rows represent taxa, columns represent individual samples and are colored by Abx or No Abx, and the color intensity indicates CLR transformed abundance. **(D)** Forest plot of differential taxa abundances, showing the estimated differences in CLR transformed abundance between exposure to antibiotics and no exposure to antibiotics. Error bars indicate 95% confidence intervals, and taxa are ordered by the significance of difference (FDR < 0.05, BH adjustment).

We evaluated the alpha diversity changes after antibiotic administration via a linear mixed-effects model. Alpha diversity, as measured by the Shannon index, is represented over time in Figure 1A. Antibiotic usage was significantly associated with a decreased microbial diversity after considering serial fecal specimen collections from the same recipients and controlling for specimen collection time, female sex, deceased donor transplantation, preoperative cefazolin prophylaxis, and TMP-SMX PJP prophylaxis (estimated decrease in alpha diversity = −0.249, *p*-value < 0.001, Supplementary Table S4).

Beta diversity, as measured by the Bray-Curtis dissimilarity and generalized UniFrac (α = 0.5), is represented in Figure 1B. The gut microbiome composition of the Abx Group was not significantly different from the No Abx Group using Bray-Curtis and the VSAT test while considering serial specimens from the same recipients and controlling for specimen collection time, female sex, deceased donor transplantation, preoperative cefazolin prophylaxis, and TMP-SMX PJP prophylaxis (*p*-value = 0.275). However, there was a significant shift when the beta diversity incorporated the phylogenetic information using generalized UniFrac (α = 0.5) (*p*-value = 0.045, Figure 1B). This ambiguity in overall composition shift confirmed the necessity to explore the details—individual differentially abundant taxa after antibiotic exposure.

### Changes in the gut microbiota after antibiotics administration

We evaluated the impact of antibiotic administration on the gut microbiota over time. A heatmap of the 20 most common genera in the study cohort is represented in Figure 1C, with the specimens annotated by their respective exposure to antibiotics or no exposure to antibiotics. To analyze the effect of antibiotics on each of the taxa, we utilized linear mixed-effects models to regress the CLR transformed taxa abundances on the antibiotic exposure, considering serial specimens from the same recipients and controlling for specimen collection time, female sex, deceased donor transplantation, preoperative cefazolin prophylaxis, and TMP-SMX PJP prophylaxis. Antibiotics were associated with a decreased abundance of 11 taxa (*Anaerostipes, Coprococcus, Ruminococcus, Alistipes, Lutispora, Dorea, Eubacterium, Erysipelotrichaceae*-Unspecified*, Anaerovorax, Parabacteroides, Lachnospiraceae*-Unspecified) and increased abundance of 16 taxa *(Staphylococcus, Scardovia, Parascardovia, Atopobium, Enterococcus, Propionibacterium, Corynebacterium, Leuconostoc, Lactobacillus, Granulicatella, Vallitalea, Rothia, Olsenella, Howadrella, Oribacterium,* and *Solobacterium*) (FDR < 0.05). The estimated differences in abundance between the Abx and No Abx Groups, along with confidence intervals, are represented in Figure 1D and Supplementary Table S5.

### Different class of antibiotics associated with decreased gut microbial diversity

**Figure 2:**
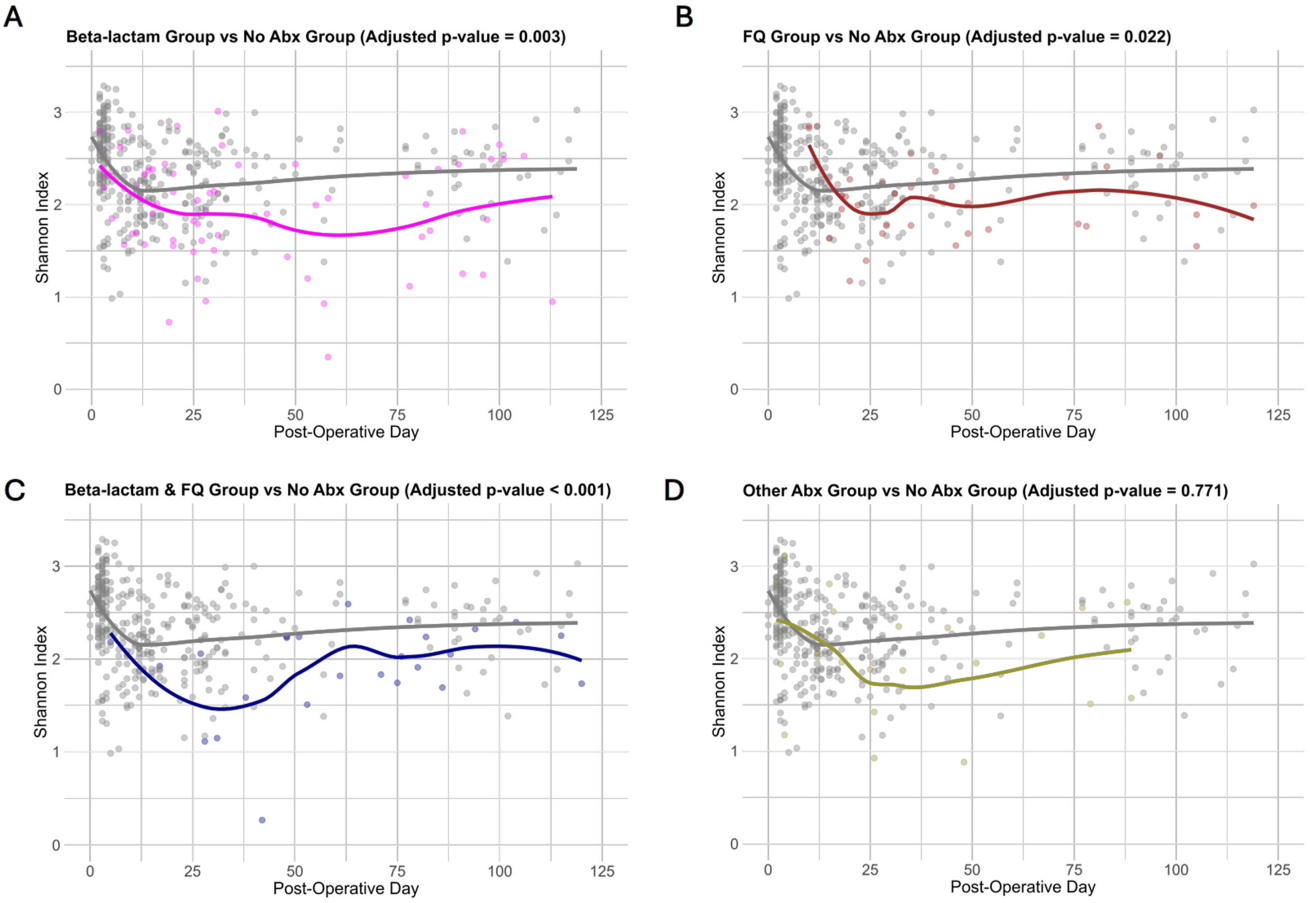
Changes in microbial diversity across antibiotics subgroups. **(A–D)** Shannon index is represented on the *y* axis and post-operative day is on the *x* axis. Each point represents a fecal specimen with color showing exposure to antibiotics with the fitted line indicating the overall trend over time. The plots illustrate differences in the Shannon index over time between exposure to Beta-lactam **(A)**, exposure to FQ **(B)**, exposure to Beta-lactam & FQ **(C)**, and exposure to Other antibiotics **(D)**, against exposure to no antibiotics. Adjusted *p*-value (Bonferroni adjustment) is shown above each figure.

Given that different subclasses of antibiotics target different bacteria, we next investigated the effects of the subgroups of antibiotics on the gut microbiota. Shannon index is shown over time, grouped by classes of antibiotics: exposure to Beta-lactam, exposure to FQ, exposure to Beta-lactam & FQ, and exposure to other antibiotics (Figure 2A–D). Using the linear mixed-effects model, which considered serial specimens from the same recipients and controlled for specimen collection time, female sex, deceased donor transplantation, preoperative cefazolin prophylaxis, and TMP-SMX PJP prophylaxis, we showed that exposure to Beta-lactam antibiotics (adjusted *p*-value = 0.003), FQ antibiotics (adjusted *p*-value = 0.022), and Beta-lactam & FQ antibiotics (adjusted *p*-value < 0.001), but not Other antibiotics (adjusted *p*-value = 0.771) were associated with decreased microbial diversity as compared to the No Abx Group (Supplementary Table S6).

### Differential changes by antibiotic classes on the gut microbiota

**Figure 3:**
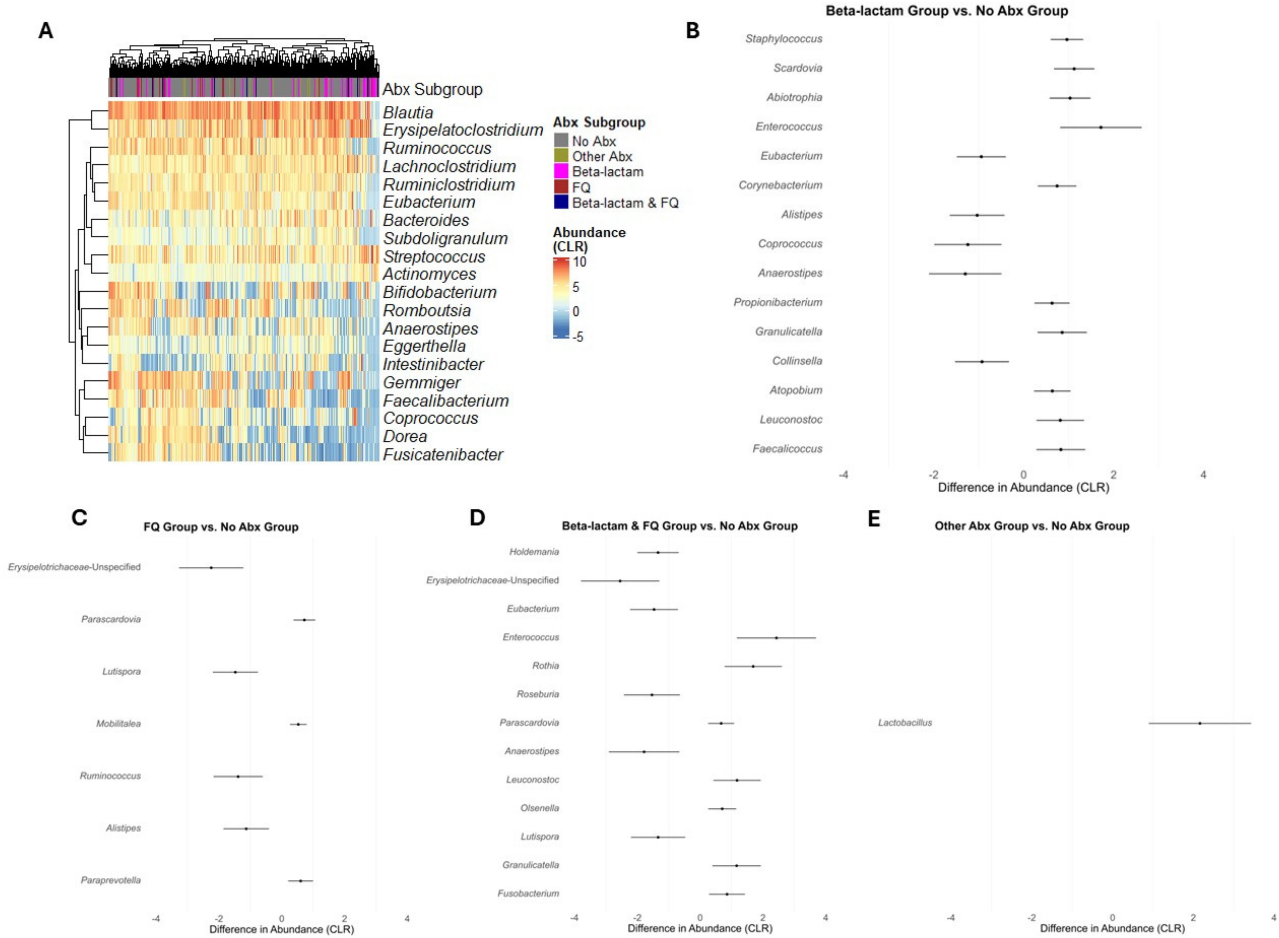
Differential abundance analysis for taxa between exposure to different antibiotics. **(A)** Heatmap of CLR transformed abundances of the 20 most common taxa, clustered by hierarchical grouping across the exposure to the 4 antibiotics subgroup and exposure to no antibiotics. Rows represent taxa, columns represent individual samples and are colored by the antibiotic subgrouping, and the color intensity indicates abundance. **(B–E)** Forest plots representing the estimated differences in CLR transformed abundances between exposure to the subclasses of antibiotics (Beta-lactam, FQ, Beta-lactam & FQ, and Other Abx) and exposure to no antibiotics (No Abx). Error bars indicate 95% confidence intervals, and taxa are ordered by the significance of difference (FDR < 0.05, BH adjustment).

**Figure 4:**
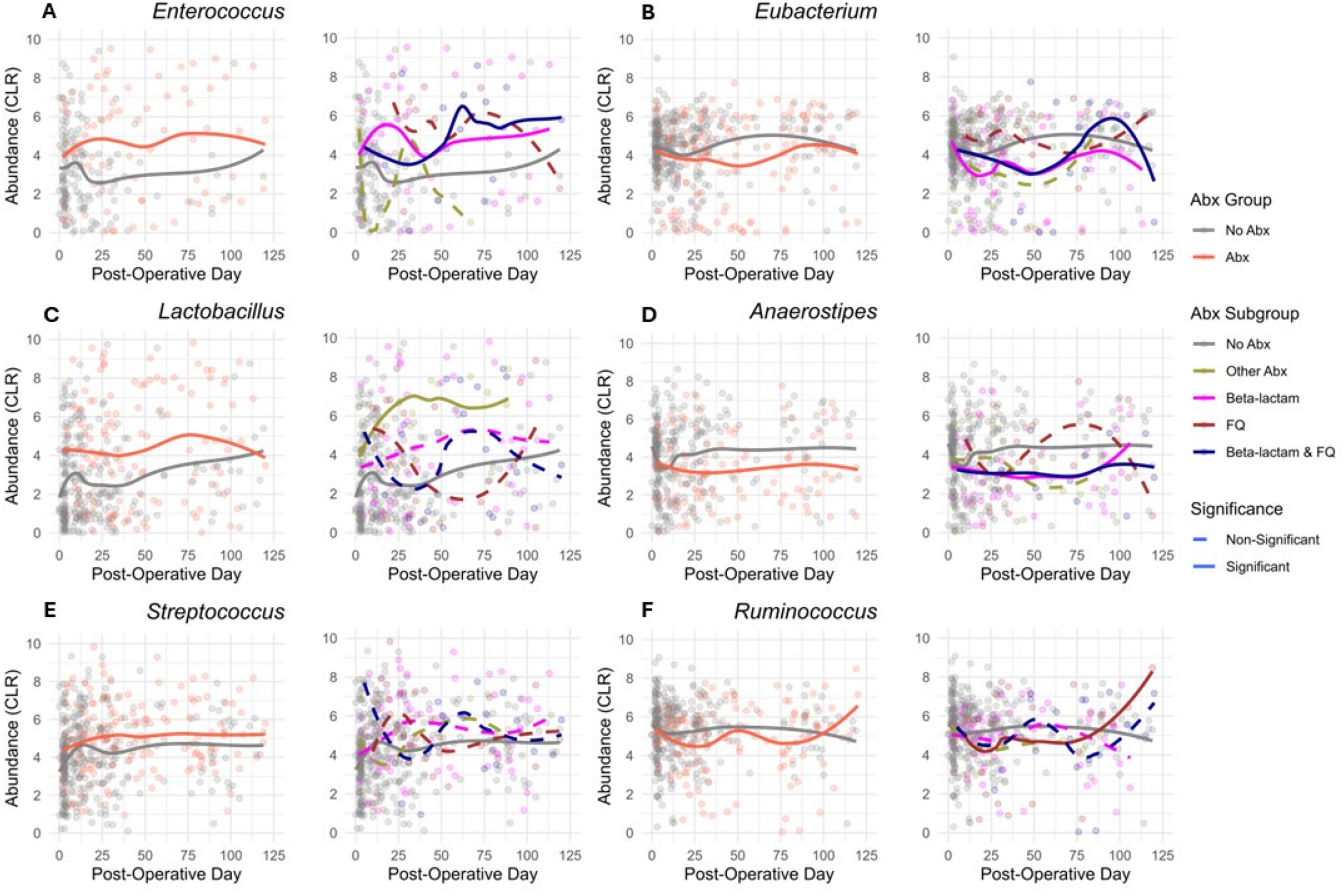
Temporal dynamics of common abundant taxa. **(A–F)** Comparative CLR abundance profiles of taxa over post-operative days. For each taxon, the left plot displays the two-group comparison (Abx vs. No Abx), while the right plot examines the variation across 5 antibiotic subgroups: Beta-lactam, FQ, Beta-lactam & FQ, No Abx, and Other Abx. The taxa analyzed are **(A)** *Enterococcus*, **(B)** *Eubacterium*, **(C)** *Lactobacillus*, **(D)** *Anaerostipes*, **(E)** *Streptococcus*, and **(F)** *Ruminococcus*. Solid lines indicate a significant difference (FDR < 0.05, BH adjustment), while dashed lines represent non-significant differences (FDR > 0.05, BH adjustment) in the abundance of corresponding group compared to the No Abx exposure.

We next evaluated whether subclasses of antibiotics were associated with differential changes in gut microbial abundances. A heatmap of the 20 most common genera in the study cohort is represented in Figure 3A, grouped by the exposure to Beta-lactam antibiotics, FQ antibiotics, Beta-lactam & FQ antibiotics, Other antibiotics, and no antibiotics. To analyze the effects of specific antibiotics, we utilized linear mixed-effects models to regress the CLR transformed abundance of each taxon on the antibiotic exposure involving the 5 groups, considering serial specimens from the same recipients and controlling for specimen collection time, female sex, deceased donor transplantation, preoperative cefazolin prophylaxis, and TMP-SMX PJP prophylaxis. Compared to exposure to no antibiotics, exposure to Beta-lactam antibiotics was associated with decreases in the abundance of 5 taxa (*Eubacterium, Alistipes, Coprococcus, Anaerostipes,* and *Collinsella*) and increases in the abundance of 10 taxa (*Staphylococcus, Scardovia, Abiotrophia, Enterococcus, Corynebacterium, Propionibacterium, Granulicatella, Atopobium, Leuconostoc, and Faecalicoccus*) (Figure 3B and Supplementary Table S7A). Compared to exposure to no antibiotics, exposure to FQ was associated with a decreased abundance of 4 taxa (*Erysipelotrichaceae*-Unspecified, *Lutispora, Ruminococcus,* and *Alistipes)* and an increased abundance of 3 taxa (*Parascardovia, Mobilitalea,* and *Paraprevotella*) (Figure 3C and Supplementary Table S7B). Compared to exposure to no antibiotics, exposure to Beta-lactam & FQ antibiotics was associated with a decreased abundance of 6 taxa (*Holdemania, Erysipelotrichaceae*-Unspecified, *Eubacterium, Roseburia, Anaerostipes,* and *Lutispora*) and increased abundance of 7 taxa (*Enterococcus, Rothia, Parascardovia, Leuconostoc, Olsenella, Granulicatella,* and *Fusobacterium*) (Figure 3D and Supplementary Table S7C). Compared to exposure to no antibiotics, exposure to other antibiotics was associated with an increased abundance of *Lactobacillus* (Figure 3E and Supplementary Table S7D). Temporal dynamics of common taxa that were significantly changed after antibiotic exposure are represented in graphs by antibiotic subgroups (Figure 4A–F).

Since other antibiotics could influence the gut microbiota beyond the respective groups, we also performed the linear mixed-effects regression analysis removing 39 kidney transplant recipients who ever received other antibiotics, allowing us to analyze 4 groups instead of 5 groups: Beta-lactam antibiotics, FQ antibiotics, Beta-lactam & FQ antibiotics, and no antibiotics. Supplementary Table S8 shows the top 10 taxa that were different between exposure to Beta-lactam antibiotics and exposure to no antibiotics (Supplementary Table S8A), between exposure to FQ antibiotics and exposure to no antibiotics (Supplementary Table S8B), and between exposure to Beta-lactam & FQ antibiotics and exposure to no antibiotics (Supplementary Table S8C). Many of these taxa overlapped with the respective table in Supplementary Table S7. However, because the sample size was reduced, all taxa had FDR > 0.05, with several having FDR < 0.10 (marginally significant).

Because Beta-lactam group is a heterogeneous group, we did a further subgroup analysis restricted to 19 patients who received the Beta-lactam antibiotics alone and divided them into narrow-spectrum Beta-lactams and broad-spectrum Beta-lactam with the classification included in Supplementary Table S1. Supplementary Table S9 lists the top 10 taxa that were different between broad-spectrum Beta-lactam antibiotics and narrow-spectrum Beta-lactam antibiotics. In this small subset of 19 kidney transplant recipients, we did not find significantly different taxa between the 2 groups (FDR > 0.05).

## Discussion

Our study provides one of the first comprehensive descriptions of the impact of antibiotics subclasses on the gut microbiota in the immunocompromised kidney transplant population. We report differential effects of the antibiotics on the gut abundance of disease-associated bacteria such as *Enterococcus* and *Staphylococcus* and on commensal bacteria such as *Ruminococcus* and *Eubacterium.* Importantly, our study highlights distinct gut microbial changes after antibiotic administration when compared to non-immunosuppressed populations*.* In an elegant systemic review of the effect of antibiotics on gut microbial composition, Zimmermann et al. reviewed 129 studies involving 2076 participants and 301 controls, importantly excluding studies involving immunosuppressed patients (7). Compared to our data, cephalosporins were associated with increases in the gut abundance of *Enterococcus,* consistent with beta-lactam's association with increases in the same bacterial taxa in our dataset. In contrast, our data noted decreases in particular abundances of commensal bacterial taxa such as *Coprococcus, Alistipes,* and *Anerostipes,* which we have previously identified as associated with post-transplant diarrhea (8). Our data with fluoroquinolones are also consistent with the Zimmermann et al. study in that fluoroquinolones were associated with decreased gut abundance of *Ruminococcus* in both studies*.* With respect to immunosuppressed populations other than kidney transplant recipients, data also support that specific antibiotic classes differentially affect the gut microbiota. In an allogenic bone marrow transplant population, metronidazole was associated with an increased domination of *Enterococcus* (2). In a liver transplant study, exposure to beta-lactams, glyco-/lipo-peptides, or carbapenems was associated with decreased gut microbial diversity (9). Differences in starting gut microbiota likely explain the differential effects of antibiotics on the gut microbiota in different immunosuppressed populations.

The current investigation significantly extends our earlier analysis on the role of antibiotics on the gut abundance of *Escherichia* and *Enterococcus* (4). Specifically, we expand the analysis to account for the impact of antibiotics and evaluate the full set of taxa at the genera level. In addition, we utilize mixed-effects models to account for the repeated collection of specimens from the same recipients as well as the timing of the specimens. Our study highlights that the group of beta-lactam antibiotics was associated with an increased gut abundance of *Enterococcus* and *Staphylococcus* after antibiotic administration. Given that *Enterococcus* is resistant to cephalosporin antibiotics (10), our data are inherently consistent with such changes in gut abundance in the kidney transplant population. Additionally, increased gut abundance of *Enterococcus* has been associated with an increased risk of *Enterococcus* infections in several studies (2, 4). Studies have also linked gut strains of *Staphylococcus* to *Staphylococcus* infectious complications. A study by Ami Bhatt et al. performed deep strain analysis on paired fecal specimens and bloodstream isolates and found a link between *Staphylococcus* in the stool and the *Staphylococcus* bloodstream isolate in the same subject (11). Our data thus highlight the potential impact of beta-lactam antibiotics on increasing the gut abundance of *Enterococcus* and *Staphylococcus,* which may place patients at a heightened risk for future infectious complications caused by these bacteria. In contrast, we did not find a significant effect of fluoroquinolones on the abundance of these specific bacteria. A potential explanation is that *Enterococcus* and *Staphylococcus* are sensitive to fluoroquinolones.

Our study also highlights the differential effects of antibiotic classes on common gut commensal bacteria. We find that the beta-lactam group of antibiotics decreases the gut abundance of *Eubacterium, Coprococcus, Anaerostipes,* and *Alistipes*, while the fluoroquinolone group of antibiotics decreases the gut abundance of *Ruminococcus* and *Alistipes.* Many of these gut bacteria produce short-chain fatty acids (SCFAs) such as butyrate, which are linked to gut health and colon inflammation (12). *Eubacterium* are a group of gram-positive obligate anaerobic bacteria that produce butyrate and propionate (13). *Eubacterium* has been positively associated with butyrate and increased insulin sensitivity (13). *Coprococcus* and *Anaerostipes* have also been associated with butyrate production (14–17). Several studies have also linked the gut abundance of *Ruminococcus* to butyrate levels in the gut (18). Together many of the bacteria contribute to colonic health. Decreased gut abundance of many of these bacteria can lead to colonic dysfunction. Indeed, one of the most common complaints after a kidney transplant is gastrointestinal disturbance such as diarrhea (19). Post-transplant diarrhea is common, but its etiology is not clear, with about 80% of the etiology being non-infectious (20). The etiology is commonly attributed to mycophenolate mofetil, which is a drug associated with gastrointestinal disturbances. In a subset analysis of kidney transplant recipients using this data set, we previously discovered a decreased gut abundance of 13 commensal gut bacteria associated with post-transplant diarrhea, including *Eubacterium, Coprococcus, Anaerostipes,* and *Ruminococcus* (8). Given that antibiotics are frequently given after transplantation for prophylaxis and infections, these changes in the gut microbiota may be part of the multi-factorial causation of post-transplant diarrhea.

In several prior studies using this cohort, we have identified the importance of many of the gut bacteria detected as significantly changed by antibiotic usage. Using the same cohort of 168 kidney transplant recipients, we have identified that the gut abundances of *Escherichia* and *Enterococcus* were associated with the development of *Escherichia* and *Enterococcus* bacteriuria, respectively (4). We have also identified that the combined gut abundance of *Faecalibacterium* and *Romboutsia* was associated with less development of *Enterobacteriaceae* bacteriuria (21). Many of the gut commensal bacteria taxa such as *Ruminococcus, Coprococcus, Alistipes,* and *Anaerostipes* were found to be decreased in kidney transplant recipients with post-transplant diarrhea in a subset analysis of the cohort (8). In another subset analysis, we also describe a decreased microbial diversity as associated with *Clostridioides difficile* colonization early after transplantation (22). All of our studies together suggest that lack of beneficial effects of commensal gut bacteria are associated with adverse outcomes like post-transplant diarrhea and bacteriuria. Our study thus highlights specific changes of important gut bacteria after antibiotic usage and suggest that antibiotic may not only impact colonic health but also potentially be associated with post-transplant complications. As shown in an elegant study by Swarte et al., gut microbial dysbiosis was linked to mortality after solid organ transplantation (23), highlighting the importance of the composition of the gut microbiota.

In addition, the gut microbiota may also have an impact on the immune system. In transplantation models, elegant mouse studies have elucidated the mechanisms by which the gut microbiota can influence the immune system in transplantation. Using a skin graft mouse transplantation model, the Alegre group reported that antibiotic treatment of both donor and recipient led to increased allograft survival (24), with a follow-up study showing that *Staphylococcus epidermiditis* colonization was associated with increased allograft rejection (25). The Bromberg group has reported that gut microbiota-dependent modulation of the immune system had an impact on cardiac allograft outcomes in a mouse model of heart transplantation (26). The Chadban group reported that intraperitoneal injection of the SCFA acetate with oral supplementation led to a decreased tubulitis score in a kidney mouse allograft model (27). Given that changes in the gut microbiota could be associated with immune system modulation, our study highlights the potential for antibiotics to disrupt the gut microbiota and to impact not only colonic health but also the host anti-allograft repertoire.

We highlight some limitations of our study. First, our study only utilized 16S rRNA gene sequencing that does not provide resolution at the species or strain level (28), which limits the interpretation of our data and does not allow us to evaluate differential impact on the species level. Also, some of the kidney transplant recipients received multiple antibiotics, different forms of administration such as oral and intravenous vancomycin, and different antibiotic course lengths, which were not accounted for, all of which may confound some of our results. Kidney transplant recipients are on multiple immunosuppressive agents, and mouse studies have shown an impact of the immunosuppressants on the gut microbiota (29). While our study did not control for the immunosuppressants, almost all of the kidney transplant recipients were on the standard tacrolimus/mycophenolic mofetil immunosuppression protocol. Our study did not evaluate other cofounders that could impact the gut microbiota such as acute cellular rejection, antibody mediated rejection, tacrolimus pharmocokinetics, post-transplant diarrhea, the presence of infection itself, diet on the gut microbiota, and history of antibiotic usage. For example, diet is known to affect the gut microbiota (30, 31) and a detailed diet history may explain variations in our antibiotic data results. A history of antibiotic usage prior to transplantation may also allow for a further understanding of why some kidney transplant recipients may respond differently to antibiotic usage. Future studies are needed to better define the role of these confounder on the gut microbiota in kidney transplantation.

In summary, we report that antibiotic classes have differential impacts on the gut microbiota in kidney transplant recipients. Our study reveals that antibiotics can be associated with increased gut abundances of disease-associated pathogens as well as with decreased gut abundances of commensal gut bacteria. Our data suggest that hopefully we can begin to provide more personalized medicine to kidney transplant recipients with respect to antibiotic usage. For example, if we are able to profile the gut microbiota in kidney transplant recipients in real time, we may be able to preferentially select antibiotic classes (fluoroquinolones vs. cephalosporins) to minimize the effects on the gut microbiota, assuming that they do not have known antibiotic resistance patterns of infections. In addition, after antibiotics are administered, our data suggest that we can potentially administer designer probiotics to replace the specific gut commensal microbiota based upon antibiotic classes. Currently, therapies have focused on the usage of fecal microbiota transplantation but can carry the risk of transmission of unexpected infections from multidrug resistant bacteria (32). Administering consortiums of specific gut commensal bacteria may provide an improved method of restoring gut health. With the recent work showing the gut microbiota's association with mortality in transplant recipients (23), repletion of gut microbiota may provide novel therapeutics for improving outcomes in transplant recipients.

## Data Availability

The datasets presented in this study can be found in online repositories. The names of the repository/repositories and accession number(s) can be found below: https://www.ncbi.nlm.nih.gov/gap/, phs001879.v1.p1. Local institutional board approval will be needed to access the data.

## References

[1] Dethlefsen L Huse S Sogin ML Relman DA. The pervasive effects of an antibiotic on the human gut microbiota, as revealed by deep 16S rRNA sequencing. PLoS Biol. (2008) 6(11):e280. 10.1371/journal.pbio.0060280 19018661 PMC2586385

[2] Taur Y Xavier JB Lipuma L Ubeda C Goldberg J Gobourne A Intestinal domination and the risk of bacteremia in patients undergoing allogeneic hematopoietic stem cell transplantation. Clin Infect Dis. (2012) 55(7):905–14. 10.1093/cid/cis580 22718773 PMC3657523

[3] Stoma I Littmann ER Peled JU Giralt S van den Brink MRM Pamer EG Compositional flux within the intestinal Microbiota and risk for bloodstream infection with gram-negative bacteria. Clin Infect Dis. (2021) 73(11):e4627–e35. 10.1093/cid/ciaa068 31976518 PMC8662789

[4] Magruder M Sholi AN Gong C Zhang L Edusei E Huang J Gut uropathogen abundance is a risk factor for development of bacteriuria and urinary tract infection. Nat Commun. (2019) 10(1):5521. 10.1038/s41467-019-13467-w 31797927 PMC6893017

[5] Martin-Fernandez J-A Hron K Templ M Filzmoser P Palarea-Albaladejo J. Bayesian-multiplicative treatment of count zeros in compositional data sets. Stat Modelling. (2014) 15(2):134–58. 10.1177/1471082X14535524

[6] Aitchison J. The statistical analysis of compositional data. J Royal Stat Soc Series B (Methodological). (1982) 44:139–60. 10.1111/j.2517-6161.1982.tb01195.x

[7] Zimmermann P Curtis N. The effect of antibiotics on the composition of the intestinal microbiota—a systematic review. J Infect. (2019) 79(6):471–89. 10.1016/j.jinf.2019.10.008 31629863

[8] Lee JR Magruder M Zhang L Westblade LF Satlin MJ Robertson A Gut microbiota dysbiosis and diarrhea in kidney transplant recipients. Am J Transplant. (2019) 19(2):488–500. 10.1111/ajt.14974 29920927 PMC6301138

[9] Annavajhala MK Gomez-Simmonds A Macesic N Sullivan SB Kress A Khan SD Colonizing multidrug-resistant bacteria and the longitudinal evolution of the intestinal microbiome after liver transplantation. Nat Commun. (2019) 10(1):4715. 10.1038/s41467-019-12633-4 31624266 PMC6797753

[10] Shepard BD Gilmore MS. Antibiotic-resistant enterococci: the mechanisms and dynamics of drug introduction and resistance. Microbes Infect. (2002) 4(2):215–24. 10.1016/S1286-4579(01)01530-1 11880055

[11] Tamburini FB Andermann TM Tkachenko E Senchyna F Banaei N Bhatt AS. Precision identification of diverse bloodstream pathogens in the gut microbiome. Nat Med. (2018) 24(12):1809–14. 10.1038/s41591-018-0202-8 30323331 PMC6289251

[12] Portincasa P Bonfrate L Vacca M De Angelis M Farella I Lanza E Gut microbiota and short chain fatty acids: implications in glucose homeostasis. Int J Mol Sci. (2022) 23(3):1105. 10.3390/ijms23031105 PMC8835596 35163038

[13] Mukherjee A Lordan C Ross RP Cotter PD. Gut microbes from the phylogenetically diverse genus Eubacterium and their various contributions to gut health. Gut Microbes. (2020) 12(1):1802866. 10.1080/19490976.2020.1802866 32835590 PMC7524325

[14] Bui TPN Schols HA Jonathan M Stams AJM de Vos WM Plugge CM. Mutual metabolic interactions in co-cultures of the intestinal Anaerostipes rhamnosivorans with an acetogen, methanogen, or pectin-degrader affecting butyrate production. Front Microbiol. (2019) 10:2449. 10.3389/fmicb.2019.02449 31736896 PMC6839446

[15] Lee JY Kang W Shin NR Hyun DW Kim PS Kim HS Anaerostipes hominis sp. nov., a novel butyrate-producing bacteria isolated from faeces of a patient with Crohn's disease. Int J Syst Evol Microbiol. (2021) 71(12):005129. 10.1099/ijsem.0.005129 34870576

[16] Murdoch DA. Gram-positive anaerobic cocci. Clin Microbiol Rev. (1998) 11(1):81–120. 10.1128/CMR.11.1.81 9457430 PMC121377

[17] Riviere A Selak M Lantin D Leroy F De Vuyst L. Bifidobacteria and butyrate-producing colon Bacteria: importance and strategies for their stimulation in the human gut. Front Microbiol. (2016) 7:979. 10.3389/fmicb.2016.00979 27446020 PMC4923077

[18] Sasaki M Schwab C Ramirez Garcia A Li Q Ferstl R Bersuch E The abundance of Ruminococcus bromii is associated with faecal butyrate levels and atopic dermatitis in infancy. Allergy. (2022) 77(12):3629–40. 10.1111/all.15440 35917214 PMC10087690

[19] Gioco R Corona D Ekser B Puzzo L Inserra G Pinto F Gastrointestinal complications after kidney transplantation. World J Gastroenterol. (2020) 26(38):5797–811. 10.3748/wjg.v26.i38.5797 33132635 PMC7579754

[20] Bunnapradist S Neri L Wong W Lentine KL Burroughs TE Pinsky BW Incidence and risk factors for diarrhea following kidney transplantation and association with graft loss and mortality. Am J Kidney Dis. (2008) 51(3):478–86. 10.1053/j.ajkd.2007.11.013 18295064

[21] Magruder M Edusei E Zhang L Albakry S Satlin MJ Westblade LF Gut commensal microbiota and decreased risk for Enterobacteriaceae bacteriuria and urinary tract infection. Gut Microbes. (2020) 12(1):1805281. 10.1080/19490976.2020.1805281 32865119 PMC7524266

[22] Westblade LF Satlin MJ Albakry S Botticelli B Robertson A Alston T Gastrointestinal pathogen colonization and the microbiome in asymptomatic kidney transplant recipients. Transpl Infect Dis. (2019) 21(6):e13167. 10.1111/tid.13167 31502737 PMC6917898

[23] Swarte JC Li Y Hu S Bjork JR Gacesa R Vich Vila A Gut microbiome dysbiosis is associated with increased mortality after solid organ transplantation. Sci Transl Med. (2022) 14(660):eabn7566. 10.1126/scitranslmed.abn7566 36044594

[24] Lei YM Chen L Wang Y Stefka AT Molinero LL Theriault B The composition of the microbiota modulates allograft rejection. J Clin Invest. (2016) 126(7):2736–44. 10.1172/JCI85295 27322054 PMC4922695

[25] Lei YM Sepulveda M Chen L Wang Y Pirozzolo I Theriault B Skin-restricted commensal colonization accelerates skin graft rejection. JCI Insight. (2019) 4(15):e127569. 10.1172/jci.insight.127569 PMC6693824 31310590

[26] Bromberg JS Hittle L Xiong Y Saxena V Smyth EM Li L Gut microbiota-dependent modulation of innate immunity and lymph node remodeling affects cardiac allograft outcomes. JCI Insight. (2020) 5(15):e142528. 10.1172/jci.insight.142528 32759493 PMC7455058

[27] Wu H Singer J Kwan TK Loh YW Wang C Tan J Gut microbial metabolites induce donor-specific tolerance of kidney allografts through induction of T regulatory cells by short-chain fatty acids. J Am Soc Nephrol. (2020) 31(7):1445–61. 10.1681/ASN.2019080852 32482686 PMC7350991

[28] Claesson MJ Wang Q O’Sullivan O Greene-Diniz R Cole JR Ross RP Comparison of two next-generation sequencing technologies for resolving highly complex microbiota composition using tandem variable 16S rRNA gene regions. Nucleic Acids Res. (2010) 38(22):e200. 10.1093/nar/gkq873 20880993 PMC3001100

[29] Zhang Z Liu L Tang H Jiao W Zeng S Xu Y Immunosuppressive effect of the gut microbiome altered by high-dose tacrolimus in mice. Am J Transplant. (2018) 18(7):1646–56. 10.1111/ajt.14661 29316256

[30] Scott KP Gratz SW Sheridan PO Flint HJ Duncan SH. The influence of diet on the gut microbiota. Pharmacol Res. (2013) 69(1):52–60. 10.1016/j.phrs.2012.10.020 23147033

[31] Zhang N Ju Z Zuo T. Time for food: the impact of diet on gut microbiota and human health. Nutrition. (2018):51–2:80–5. 10.1016/j.nut.2017.12.005 29621737

[32] DeFilipp Z Bloom PP Torres Soto M Mansour MK Sater MRA Huntley MH Drug-resistant E. coli bacteremia transmitted by fecal microbiota transplant. N Engl J Med. (2019) 381(21):2043–50. 10.1056/NEJMoa1910437 31665575

