# Supplementary material for: Antibiotic subclasses differentially perturb the gut microbiota in kidney transplant recipients
Source: Front Transplant. 2024 Sep 20;3:1400067. doi: 10.3389/frtra.2024.1400067 (PMC11451434; doi:10.3389/frtra.2024.1400067)
Supplement: Supplementary file 1 [file Datasheet1.pdf]

Supplemental Methods

Patient Cohort

The study cohort for this investigation are the 168 kidney transplant recipients who were previously recruited for serial fecal specimen profiling from August 2015 to November 2016. A total of 510 fecal specimens were collected. Demographical and transplant related characteristics as well as antibiotic usage were obtained. The Weill Cornell Institutional Review Board approved the study and all patients provided written informed consent.

Gut Microbiota Profiling of Fecal Specimens

16S rRNA gene sequencing of the V4-V5 hypervariable region was performed for each of the 510 fecal specimens as previously reported (1). In brief, DNA from fecal specimens was extracted by pheno-chloroform extraction with bead-beater sonication and purified using QIAamp mini spin columns (1). The 16S rRNA gene V4-V5 region was amplified using a unique 12-base Golay barcode preceding primers (1). The PCR fragments were sequenced using an Illumina MiSeq (250 base pair by 250 base pair) (1).

Taxonomic assignment of the 16s rRNA genes was then performed as previously described (1). Sequences were run with the UPARSE pipeline (2) (to remove chimeric sequences and group into operational taxonomic units). A custom Python script was used for taxonomic assignment using BLAST (3) (NCBI Refseq as reference training set).

Statistical Analyses

Categorical variables between the Abx Group and No Abx Group at the patient level were compared using Fisher's exact test, and continuous variables were compared using the Wilcoxon rank sum test (Kruskal-Wallis test for multi-group comparisons). Transplant characteristics with a p-value less than 0.05 were adjusted as covariates in the mixed-effects regression models.

We conducted community-level analysis (alpha and beta diversity analysis) and individual-level analysis (differential abundance analysis) via mixed-effects regression models. These statistical analyses were longitudinal in nature, analyzing time-specific outcomes (microbial feature, microbial profile, or individual taxa) given both time-invariant (e.g., female sex) and time-specific (e.g., antibiotic use) exposures while considering within-subject correlations.

For alpha diversity, we utilized the Shannon index (computed based on relative abundances). We conducted linear mixed-effects regression that regressed alpha diversity of all the specimens on the time-ever antibiotics use, considering the serial collection of specimens from the same subjects and adjusting for post-operative days, female sex, deceased donor transplantation, preoperative antibiotic cefazolin prophylaxis, and PJP trimethoprim/sulfamethoxazole (TMP/SMX) prophylaxis. A p-value less than 0.05 was considered significant. We also executed the model using the antibiotic subgroups for pairwise comparisons between each of the 4 Abx Groups and No Abx Group. Bonferroni correction was

utilized to control the family-wise error rate, and an adjusted p-value less than 0.05 was considered significant.

For beta diversity, we utilized Bray-Curtis dissimilarity and generalized UniFrac (with the weight on abundant lineages α = 0.5, a commonly used robust weight) dissimilarity (9) that incorporates phylogenetic distances (both computed based on relative abundances). Principal coordinate analysis (PCoA) plots were used to visualize the fecal specimens stratified by antibiotic exposure and no antibiotic exposure. We note that the plot neither incorporates the within-subject correlations nor adjusts for covariates and was used for visual presentation only. Numerically, VSAT (4), a longitudinal extension of PERMANOVA (5) and MiRKAT (6) that considers the within-subject correlations, was used to test the overall association between antibiotic use and the gut microbiome composition, controlling for the same set of covariates that were significantly different at the patient level. A p-value less than 0.05 was considered significant.

To identify differentially abundant taxa, we used linear mixed-effects models to regress the CLR transformed abundances of each taxon on the time-ever antibiotics use while adjusting for the same set of covariates that were significantly different at the patient level. Benjamini-Hochberg (BH) procedure was employed to adjust the resulting p-values. When we executed the model using the antibiotic subgroups, p-values of the 4 pairwise comparisons across all taxa were adjusted together. A false discovery rate (FDR) less than 0.05 was considered significant.

Supplemental Flowchart

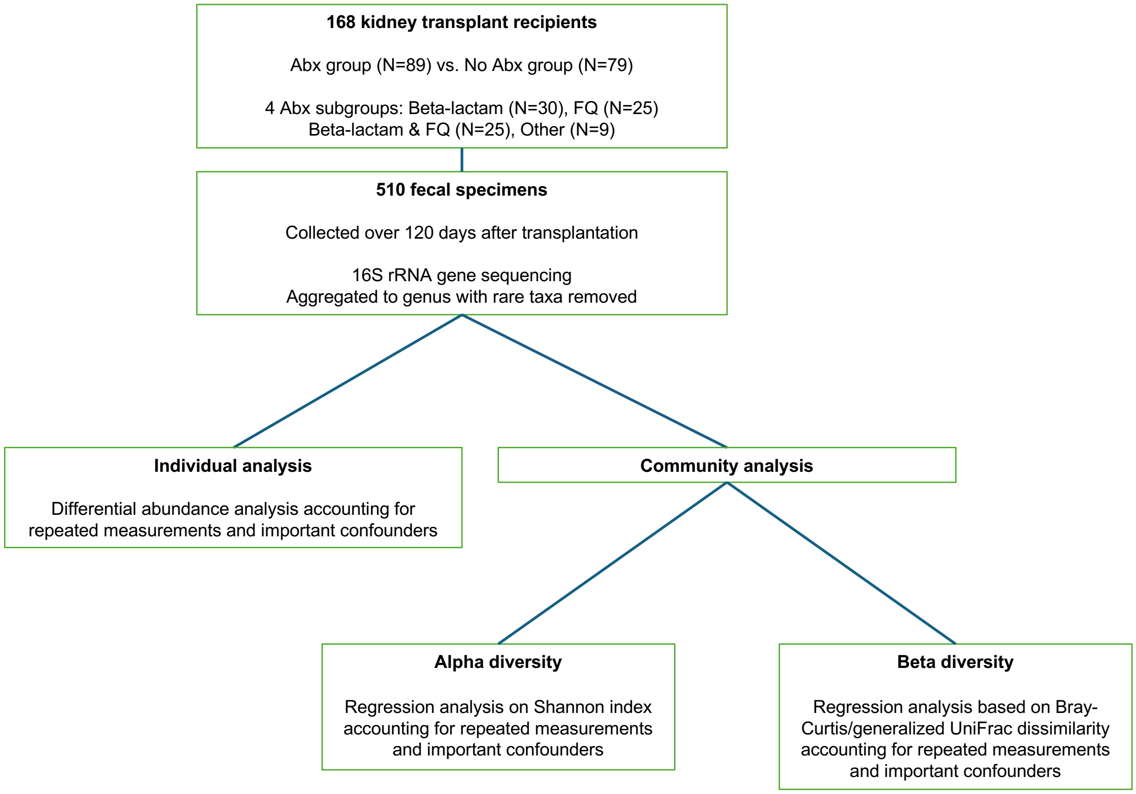

Supplemental Glossary

Terms	Explanation
Imputation	The process of replacing missing data with substituted values to achieve more robust analysis
Geometric Bayesian multiplicative replacement method	A Bayesian-multiplicative method to impute zeros in compositional count data
Normalization	A pre-processing step to remove non-biological variation, achieving comparable microbiome data
Centered log-ratio transformation	A normalization method for compositional data (constraint: sum up to a constant total), allowing for standard statistical analysis that assume independence
Fisher's exact test	A statistical significance test used in the analysis of crosstabs for discrete variables, particularly useful when sample sizes are not big
Wilcoxon rank sum test	A statistical significance test used to compare the medians of two independent groups, robust as it is a nonparametric method
Mixed-effects regression models	A type of statistical model that incorporates both fixed effects that have a consistent effect across individuals

	and random effect that might affect each individual differently, particularly useful in situations where data are collected repeatedly over time
Bonferroni correction	A statistical method used to address the problem of multiple comparisons, designed to control the family-wise error rate
Family-wise error rate	The probability of making at least one type I error when performing multiple hypothesis tests
Bray-Curtis dissimilarity	A statistic used to quantify the dissimilarity in species composition between two different samples, based on counts at each sample
Generalized UniFrac dissimilarity	A statistic to compare microbial communities, considering phylogenetic distances between observed organisms and using a parameter α to adjust weight on abundant lineages
Principal coordinate analysis	A method used to visualize and analyze the relationships between microbiota samples based on their distances (particularly non-Euclidean, to explore patterns in composition, uncovering structure or clusters
VSAT	Variant-set association test for mixed-effects models, a longitudinal extension of PERMANOVA and MiRKAT
PERMANOVA	Permutational Multivariate Analysis of Variance, a non-parametric statistical test to compare groups based on their microbiome composition (centroids + dispersion)
MiRKAT	Microbiome Regression-Based Kernel Association Test, a statistical method to assess the association between microbiome composition and various host traits or environmental factors
Benjamini-Hochberg procedure	A statistical method used to control the false discovery rate when conducting multiple hypothesis tests, particularly useful in omics analysis
False discovery rate	The proportion of false positives among all rejected hypotheses, particularly relevant in omics fields

References

1. Magruder M, Sholi AN, Gong C, Zhang L, Edusei E, Huang J, et al. Gut uropathogen abundance is a risk factor for development of bacteriuria and urinary tract infection. *Nat Commun.* 2019;10(1):5521.
2. Edgar RC. MUSCLE: multiple sequence alignment with high accuracy and high throughput. *Nucleic Acids Res.* 2004;32(5):1792-7.
3. Altschul SF, Gish W, Miller W, Myers EW, and Lipman DJ. Basic local alignment search tool. *J Mol Biol.* 1990;215(3):403-10.
4. Zhan X, Banerjee K, and Chen J. Variant-set association test for generalized linear mixed model. *Genet Epidemiol.* 2021;45(4):402-12.
5. Anderson MJ. Permutational Multivariate Analysis of Variance (PERMANOVA). *Genet Epidemiol.* 2017;45(4):402-12.
6. Zhao N, Chen J, Carroll IM, Ringel-Kulka T, Epstein MP, Zhou H, et al. Testing in Microbiome-Profiling Studies with MiRKAT, the Microbiome Regression-Based Kernel Association Test. *Am J Hum Genet.* 2015;96(5):797-807.

Supplemental Tables

Supplement Table 1. List of antibiotics by categories.

Classes	Antibiotics
Beta-lactams	piperacillin tazobactam (broad)
	amoxicillin (narrow)
	ampicillin sulbactam (broad)
	amoxicillin clavulanate (narrow)
	ampicillin (narrow)
	penicillin v potassium (narrow)
	ceftriaxone (broad)
	cefpodoxime (narrow)
	cefazolin (narrow)
	cephalexin (narrow)
	cefepime (broad)
	ceftolozane (broad)
	ceftolozane/tazobactam (broad)
	cefadroxil (narrow)
	meropenem (broad)
	ertapenem (broad)
	aztreonam (broad)
Fluoroquinolones	levofloxacin
	ciprofloxacin
Other	vancomycin
	metronidazole
	isoniazid
	linezolid
	azithromycin
	trimethoprim
	nitrofurantoin
	doxycycline
	clindamycin
	amikacin
	daptomycin
	erythromycin
	fosfomycin

Supplemental Table 2a. Characteristics of Abx and No Abx Groups at the recipient level.

	Abx (n=89)	No Abx (n=79)	p-value[1]
Age	56	53	0.078
Female Sex	48 (53.9%)	28 (35.4%)	0.020
AA Race	26 (29.2%)	18 (22.8%)	0.383
Diabetes Mellitus	31 (34.8%)	18 (22.8%)	0.092
Preoperative Cefazolin Antibiotic Prophylaxis	68 (76.4%)	71 (89.9%)	0.025
TMP-SMX PJP Prophylaxis	80 (89.9%)	79 (100.0%)	0.004
Deceased Donor Transplantation	35 (39.3%)	14 (17.7%)	0.002
Prior Transplant	12 (13.5%)	12 (15.2%)	0.827
Delayed Graft Function	18 (20.2%)	10 (12.7%)	0.218
Anti-thymocyte Globulin induction	64 (71.9%)	64 (81.0%)	0.205
Prednisone Steroid Maintenance	26 (29.2%)	19 (24.1%)	0.489

[1] p-values were calculated using Wilcoxon rank sum test for continuous variables and Fisher's exact test for dichotomized variables.

Supplemental Table 2b. Characteristics of Abx Group and No Abx Group at the specimen-level.

	Abx (n=148)	No Abx (n=362)
Age	53	53
Female Sex	83 (56.1%)	141 (39.0%)
AA Race	51 (34.5%)	86 (23.8%)
Diabetes Mellitus	55 (37.2%)	98 (27.1%)
Preoperative Cefazolin Antibiotic Prophylaxis	107 (72.3%)	315 (87.0%)
TMP-SMX PJP Prophylaxis	136 (91.9%)	352 (97.2%)
Deceased Donor Transplantation	74 (50%)	81 (22.4%)
Prior Transplant	20 (13.5%)	52 (14.4%)
Delayed Graft Function	29 (19.6%)	54 (14.9%)
Anti-thymocyte Globulin induction	111 (75%)	281 (77.6%)
Prednisone Steroid Maintenance	39 (26.4%)	90 (24.9%)

Supplement Table 3a. Characteristics of the 4 Abx Subgroup and No Abx Group at the recipient-level.

	No Abx (n=79)	Other (n=9)	Beta-lactam (n=30)	FQ (n=25)	Beta-lactam & FQ (n=26)	p-value[1]
Age	53	60	52	62	53	0.131
Female Sex	28 (35.4%)	3 (33.3%)	16 (53.3%)	14 (56.0%)	15 (60.0%)	0.099
AA Race	18 (22.8%)	1 (11.1%)	11 (36.7%)	6 (24.0%)	8 (32.0%)	0.481
Diabetes Mellitus	18 (22.8%)	4 (44.4%)	11 (36.7%)	9 (36.0%)	7 (28.0%)	0.366
Preoperative Cefazolin Antibiotic Prophylaxis	71 (89.9%)	5 (55.6%)	25 (83.3%)	20 (80.0%)	18 (72.0%)	0.042
TMP-SMX PJP Prophylaxis	79 (100%)	6 (66.7%)	29 (96.7%)	21 (84.0%)	24 (96.0%)	<0.001
Deceased Donor Transplantation	14 (17.7%)	2 (22.2%)	16 (53.3%)	9 (36.0%)	8 (32.0%)	0.006
Prior Transplant	12 (15.2%)	3 (33.3%)	5 (16.7%)	1 (4.0%)	3 (12.0%)	0.252
Delayed Graft Function	10 (12.7%)	0 (0%)	7 (23.3%)	5 (20.0%)	6 (24.0%)	0.298
Anti-thymocyte Globulin induction	64 (81%)	5(55.6%)	21 (70.0%)	17 (68.0%)	21 (84.0%)	0.217
Prednisone Steroid Maintenance	19 (24.1%)	3 (33.3%)	11 (36.7%)	8 (32.0%)	4 (16.0%)	0.409

[1] p-values were calculated using Kruskal-Wallis test for continuous variables and Fisher's exact test for dichotomized variables.

Supplement Table 3b. Characteristics of 4 Abx Subgroup and No Abx Group at the specimen-level.

	No Abx (n=362)	Other (n=25)	Beta-lactam (n=63)	FQ (n=35)	Beta-lactam & FQ (n=25)
Age	53	60	50	65	53
Female Sex	141 (39.0%)	14 (56.0%)	36 (57.1%)	18 (51.4%)	15 (60.0%)
AA Race	86 (23.8%)	9 (36.0%)	26 (41.3%)	10 (28.6%)	6 (24.0%)
Diabetes Mellitus	98 (27.1%)	12 (48.0%)	21 (33.3%)	13 (37.1%)	9 (36.0%)

Preoperative Cefazolin Antibiotic Prophylaxis	315 (87.0%)	17 (68.0%)	46 (73.0%)	25 (71.4%)	19 (76.0%)
TMP-SMX PJP Prophylaxis	352 (97.2%)	19 (76.0%)	61 (96.8%)	31 (88.6%)	25 (100.0%)
Deceased Donor Transplantation	81 (22.4%)	11 (44.0%)	40 (63.5%)	15 (42.9%)	8 (32.0%)
Prior Transplant	52 (14.4%)	9 (36.0%)	7 (11.1%)	1 (2.9%)	3 (12.0%)
Delayed Graft Function	54 (14.9%)	3 (12.0%)	14 (22.2%)	7 (20.0%)	5 (20.0%)
Anti-thymocyte Globulin induction	281 (77.6%)	21 (84.0%)	43 (68.3%)	27 (77.1%)	20 (80.0%)
Prednisone Steroid Maintenance Protocol	90 (24.9%)	7 (28.0%)	24 (38.1%)	5 (14.3%)	3 (12.0%)

Supplemental Table 4. Effect estimates on alpha diversity between exposure to antibiotics and no exposure to antibiotics.

	Estimate	Lower CI	Upper CI	p-value[1]
Exposure to Antibiotics vs. No exposure	-0.249	-0.357	-0.141	<0.001
Post-Operative Day	-0.001	-0.003	0.000	0.042
Sex: Female vs. Male	-0.010	-0.121	0.102	0.866
Deceased vs. Living Donor Transplantation	-0.107	-0.231	0.016	0.087
Preoperative Cefazolin Antibiotic Prophylaxis vs. Other	-0.021	-0.171	0.128	0.781
TMP-SMX PJP Prophylaxis vs. Other	0.086	-0.179	0.351	0.522

[1] p-values were calculated using a linear mixed-effects regression.

Supplemental Table 5. Estimates on differential taxa abundance between exposure to antibiotics and no exposure to antibiotics.

	Estimate	Lower CI	Upper CI	p-value[1]	FDR[2]
Staphylococcus	0.580	0.305	0.856	<0.001	0.003
Scardovia	0.716	0.376	1.056	<0.001	0.003
Anaerostipes	-1.234	-1.838	-0.630	<0.001	0.003
Parascardovia	0.447	0.222	0.672	<0.001	0.003
Atopobium	0.603	0.299	0.908	<0.001	0.003
Coprococcus	-1.056	-1.616	-0.496	<0.001	0.004
Ruminococcus	-0.960	-1.465	-0.454	<0.001	0.004
Alistipes	-0.845	-1.304	-0.387	<0.001	0.005
Enterococcus	1.187	0.500	1.874	0.001	0.009
Lutispora	-0.813	-1.284	-0.342	0.001	0.009
Dorea	-0.956	-1.517	-0.396	0.001	0.010
Propionibacterium	0.477	0.185	0.770	0.001	0.014
Corynebacterium	0.525	0.203	0.848	0.001	0.014
Eubacterium	-0.659	-1.071	-0.246	0.002	0.015
Leuconostoc	0.633	0.235	1.031	0.002	0.015
Lactobacillus	0.985	0.371	1.599	0.002	0.015
Granulicatella	0.641	0.228	1.054	0.002	0.018
Erysipelotrichaceae-Unspecified	-1.035	-1.734	-0.337	0.004	0.026
Anaerovorax	-0.517	-0.870	-0.164	0.004	0.027
Vallitalea	0.336	0.103	0.569	0.005	0.028
Parabacteroides	-0.696	-1.183	-0.210	0.005	0.028
Rothia	0.707	0.215	1.199	0.005	0.028
Olsenella	0.338	0.105	0.571	0.005	0.028
Lachnospiraceae-Unspecified	-0.777	-1.332	-0.221	0.006	0.032
Howardella	0.286	0.076	0.496	0.008	0.038
Oribacterium	0.339	0.085	0.593	0.009	0.044
Solobacterium	0.370	0.087	0.654	0.011	0.048

[1] p-values were calculated using linear mixed-effects regressions.
[2] p-values were adjusted using Benjamini-Hochberg correction.

Supplemental Table 6. Effect estimates on alpha diversity across exposure to antibiotic subgroups compared to no exposure to antibiotics.

	Estimate	Lower CI	Upper CI	p-value[1]	Adjusted p-value[2]
Other Abx vs. No Abx	-0.146	-0.365	0.074	0.193	0.771
Beta-lactam vs. No Abx	-0.247	-0.391	-0.103	<0.001	0.003
FQ vs. No Abx	-0.243	-0.414	-0.072	0.005	0.022
Beta-lactam and FQ vs. No Abx	-0.385	-0.589	-0.182	<0.001	<0.001
Post-Operative Day	-0.001	-0.002	0.000	0.097	
Sex: Female vs. Male	-0.010	-0.122	0.102	0.865	
Deceased vs. Living Donor transplantation	-0.109	-0.234	0.015	0.086	
Preoperative Cefazolin Antibiotic Prophylaxis vs. Other	-0.022	-0.173	0.128	0.768	
TMP-SMX PJP Prophylaxis vs. Other	0.121	-0.150	0.392	0.381	

[1] p-values were calculated using a linear mixed-effects regression.
[2] p-values were adjusted using Bonferroni correction.

Supplement Table 7a. Estimates on differential taxa abundance between exposure to Beta-lactam antibiotics and no exposure to antibiotics.

	Estimate	LowerCI	UpperCI	p-value[1]	FDR[2]
Staphylococcus	0.961	0.598	1.323	<0.001	<0.001
Scardovia	1.122	0.672	1.572	<0.001	<0.001
Abiotrophia	1.030	0.577	1.484	<0.001	0.002
Enterococcus	1.716	0.809	2.622	<0.001	0.009
Eubacterium	-0.944	-1.488	-0.399	0.001	0.022
Corynebacterium	0.742	0.313	1.171	0.001	0.022
Alistipes	-1.035	-1.645	-0.426	0.001	0.024
Coprococcus	-1.242	-1.990	-0.495	0.001	0.029
Anaerostipes	-1.299	-2.105	-0.493	0.002	0.036
Propionibacterium	0.629	0.239	1.019	0.002	0.036
Granulicatella	0.855	0.306	1.403	0.002	0.039
Collinsella	-0.927	-1.525	-0.329	0.002	0.039
Atopobium	0.636	0.230	1.042	0.002	0.039
Leuconostoc	0.812	0.283	1.341	0.003	0.042
Faecalicoccus	0.825	0.282	1.368	0.003	0.044

[1] p-values were calculated using linear mixed-effects regressions.
[2] p-values were adjusted using Benjamini-Hochberg correction.

Supplement Table 7b. Estimates on differential taxa abundance between exposure to FQ antibiotics and no exposure to antibiotics.

	Estimate	LowerCI	UpperCI	p-value[1]	FDR[2]
Erysipelotrichaceae-Unspecified	-2.246	-3.270	-1.222	<0.001	0.003
Parascardovia	0.722	0.375	1.069	<0.001	0.004
Lutispora	-1.477	-2.197	-0.757	<0.001	0.004
Mobilitalea	0.528	0.261	0.795	<0.001	0.006
Ruminococcus	-1.389	-2.173	-0.605	0.001	0.019
Alistipes	-1.132	-1.856	-0.408	0.002	0.039
Paraprevotella	0.602	0.205	0.999	0.003	0.044

[1] p-values were calculated using linear mixed-effects regressions.
[2] p-values were adjusted using Benjamini-Hochberg correction.

Supplement Table 7c. Estimates on differential taxa abundance between exposure to Beta-lactam & FQ antibiotics and no exposure to antibiotics.

	Estimate	LowerCI	UpperCI	p-value[1]	FDR[2]
Holdemania	-1.342	-2.000	-0.684	<0.001	0.004

Erysipelotrichaceae-Unspecified	-2.549	-3.796	-1.301	<0.001	0.004
Eubacterium	-1.469	-2.230	-0.708	<0.001	0.008
Enterococcus	2.428	1.167	3.689	<0.001	0.008
Rothia	1.689	0.777	2.601	<0.001	0.012
Roseburia	-1.535	-2.425	-0.645	0.001	0.022
Parascardovia	0.668	0.252	1.083	0.002	0.036
Anaerostipes	-1.785	-2.908	-0.663	0.002	0.039
Leuconostoc	1.175	0.421	1.929	0.002	0.039
Olsenella	0.701	0.255	1.147	0.002	0.039
Lutispora	-1.339	-2.204	-0.475	0.002	0.039
Granulicatella	1.160	0.388	1.933	0.003	0.047
Fusobacterium	0.855	0.285	1.426	0.003	0.047

[1] p-values were calculated using linear mixed-effects regressions.
[2] p-values were adjusted using Benjamini-Hochberg correction.

Supplement Table 7d. Estimates on differential taxa abundance between exposure to Other antibiotics and no exposure to antibiotics.

	Estimate	LowerCI	UpperCI	p-value[1]	FDR[2]
Lactobacillus	2.162	0.896	3.429	<0.001	0.024

[1] p-values were calculated using linear mixed-effects regressions.
[2] p-values were adjusted using Benjamini-Hochberg correction.

Supplemental Table 8a. Estimates on differential taxa abundance between exposure to Beta-lactam antibiotics and no exposure to antibiotics, excluding patients exposed to Other antibiotics.

	Estimate	LowerCI	UpperCI	p-value[1]	FDR[2]
Faecalicoccus	1.135	0.452	1.818	0.001	**0.065**
Anaerostipes	-1.585	-2.586	-0.583	0.002	**0.083**
Butyricicoccus	0.975	0.349	1.601	0.002	**0.088**
Coprococcus	-1.253	-2.172	-0.333	0.008	0.212
Dorea	-1.184	-2.121	-0.247	0.013	0.263
Lactococcus	0.969	0.218	1.721	0.012	0.263
Oscillibacter	1.004	0.214	1.794	0.013	0.263
Catabacter	0.669	0.147	1.191	0.012	0.263
Abiotrophia	0.639	0.107	1.171	0.019	0.303
Lachnospiraceae-Unspecified	-1.120	-2.065	-0.174	0.020	0.311

[1] p-values were calculated using linear mixed-effects regressions.
[2] p-values were adjusted using Benjamini-Hochberg correction.

Supplemental Table 8b. Estimates on differential taxa abundance between exposure to FQ antibiotics and no exposure to antibiotics, excluding patients exposed to Other antibiotics.

	Estimate	LowerCI	UpperCI	p-value[1]	FDR[2]
Parascardovia	0.671	0.306	1.036	<0.001	**0.057**
Mobilitalea	0.591	0.251	0.931	0.001	**0.064**
Holdemanella	-1.075	-1.705	-0.445	0.001	**0.065**
Lutispora	-1.473	-2.361	-0.585	0.001	**0.065**
Erysipelotrichaceae-Unspecified	-1.921	-3.132	-0.710	0.002	**0.083**
Paraprevotella	0.677	0.165	1.190	0.010	0.240
Actinomyces	0.766	0.144	1.388	0.016	0.296
Anaerostipes	-1.347	-2.453	-0.241	0.017	0.303
Solobacterium	0.636	0.089	1.183	0.023	0.311
Anaerofilum	0.493	0.023	0.962	0.040	0.351

[1] p-values were calculated using linear mixed-effects regressions.
[2] p-values were adjusted using Benjamini-Hochberg correction.

Supplemental Table 8c. Estimates on differential taxa abundance between exposure to Beta-lactam & FQ antibiotics and no exposure to antibiotics, excluding patients exposed to Other antibiotics.

	Estimate	LowerCI	UpperCI	p-value[1]	FDR[2]
Eubacterium	-1.787	-2.721	-0.854	<0.001	**0.057**
Actinotignum	1.003	0.445	1.560	<0.001	**0.057**
Erysipelotrichaceae- Unspecified	-2.286	-3.939	-0.633	0.007	0.212
Parascardovia	0.672	0.177	1.167	0.008	0.212
Rothia	1.648	0.454	2.841	0.007	0.212
Olsenella	0.794	0.134	1.455	0.019	0.303
Anaerostipes	-1.724	-3.226	-0.222	0.025	0.311
Granulicatella	1.154	0.145	2.163	0.025	0.311
Atopobium	0.908	0.136	1.681	0.021	0.311
Holdemania	-0.957	-1.831	-0.083	0.032	0.349

[1] p-values were calculated using linear mixed-effects regressions.
[2] p-values were adjusted using Benjamini-Hochberg correction.

Supplemental Table 9. Estimates on differential taxa abundance between exposure to Narrow Beta-lactam antibiotics and exposure to Broad Beta-lactam antibiotics (Narrow - Broad), excluding patients exposed to FQ, Other antibiotics or not exposed to any antibiotics.

	Estimate	LowerCI	UpperCI	p-value[1]	FDR[2]
Roseburia	2.639	0.745	4.533	0.007	0.447
Acidaminococcus	1.592	0.490	2.694	0.006	0.447
Dorea	2.252	-0.087	4.591	0.059	0.677
Leuconostoc	-1.740	-3.248	-0.232	0.025	0.677
Abiotrophia	-1.326	-2.753	0.100	0.068	0.677
Enterococcus	-3.059	-5.617	-0.501	0.020	0.677
Faecalicoccus	-1.382	-2.728	-0.037	0.044	0.677
Erysipelotrichaceae-Unspecified	1.861	-0.145	3.867	0.068	0.677
Butyricimonas	0.870	-0.077	1.817	0.071	0.677
Ruminiclostridium	-1.465	-2.830	-0.101	0.036	0.677

[1] p-values were calculated using linear mixed-effects regressions.
[2] p-values were adjusted using Benjamini-Hochberg correction.
